# Streamlining First-Order Reversal Curves Analysis of Molecular Magnetism Bistability Using a Calorimetric Approach

**DOI:** 10.3390/ma18143413

**Published:** 2025-07-21

**Authors:** Diana Plesca, Cristian Enachescu, Radu Tanasa, Alexandru Stancu, Denis Morineau, Marie-Laure Boillot

**Affiliations:** 1 Faculty of Physics, “Alexandru Ioan Cuza” University, 700506 Iasi, Romania; diana.plesca@student.uaic.ro (D.P.); cristian.enachescu@uaic.ro (C.E.); alstancu@uaic.ro (A.S.); 2 Institut de Physique de Rennes, Unité Mixte de Recherche 6251, Centre National de la Recherche Scientifique, Université de Rennes, 35042 Rennes, France; denis.morineau@univ-rennes.fr; 3 Institut de Chimie Moléculaire et des Matériaux d’Orsay, Unité Mixte de Recherche 8182, Centre National de la Recherche Scientifique, Université Paris-Saclay, 91400 Orsay, France; marie-laure.boillot@universite-paris-saclay.fr

**Keywords:** spin crossover, FORC, heat capacity, calorimetry, thermogram

## Abstract

We present an alternative to the classical SQUID magnetometric measurements for the First-Order Reversal Curve (FORC) diagram approach by employing differential scanning calorimetry (DSC) experiments. After discussing the main results, the advantages and limitations of the magnetometric FORCs, we introduce the calorimetric method. We argue that, while the results are comparable to those obtained via magnetometry, the calorimetric method not only significantly simplifies the required mathematical computations but also detects subtle or overlapping phase transitions that might be hard to distinguish magnetically. The methodology is illustrated through both experimental data and mean-field simulations.

## 1. Introduction

Hysteretic spin-crossover (SCO) materials [1,2,3,4] represent a cornerstone in the advancement of functional molecular systems, offering significant potential for applications in non-volatile memory and sensing technologies [5]. Their primary advantage lies in the tunability of their switching behavior and the enhanced control they enable over cooperative interactions and environmental responsiveness, particularly when embedded in nanocomposites [6,7,8]. However, as efforts progress toward integrating these materials into functional nanodevices [9], traditional bulk measurement techniques have revealed intrinsic limitations in delivering precise, quantitative insights into microscopic switching processes, domain evolution [10], and hysteresis mechanisms. In this context, the First-Order Reversal Curves (FORC) method [11,12,13,14,15,16] has emerged as a powerful tool to overcome these challenges [17]. FORC analyses allow the decoupling of intrinsic material characteristics from extrinsic influences such as structural defects, heterogeneities, and interfacial couplings in SCO nanocomposites [18]. Over the past decade, the FORC methodology has become essential for deepening our understanding of spin-crossover phenomena [19], ultimately guiding the rational design and predictive development of next-generation bistable SCO materials for advanced nanotechnological applications [20,21,22,23].

Among the methods able to probe the spin-state switching, such as magnetometry, X-ray diffraction [24], reflectivity, and Mössbauer spectroscopy [1], calorimetry distinguishes itself as a direct and highly sensitive tool to monitor these transitions [25,26]. It is particularly valuable for identifying phase purity and multistep transitions and distinguishing between gradual and abrupt switching behaviors, even in complex systems like nanocomposites or thin films. Moreover, it is effective in revealing other phase transitions (e.g., non-magnetic) that may occur and influence the magnetic behavior, as in the case of spin-crossover (nano)materials embedded in matrices undergoing thermally induced transformations between liquid, solid, and glassy states [27]. Additionally, this approach is especially well-suited for characterizing a wide range of spin transitions under applied pressure and for verifying hydrostatic conditions. Beyond its analytical power, calorimetry also offers practical advantages, including cost-effectiveness and energy efficiency, making it a highly attractive tool in the lab investigation of SCO materials [28]. However, conventional calorimetry often struggles to resolve complex, overlapping transitions or detect subtle cooperative effects within SCO systems, primarily due to averaging effects and thermal noise [27]. When coupled with the FORC methodology, these limitations are significantly mitigated, providing access to a high-resolution map of the material’s switching dynamics.

In a previous study focused on the interplay between spin-crossover micro- and nanoparticles and their embedding matrices, we provided an initial introduction of calorimetric FORCs as a proof of concept [18]. However, that work did not delve into the technical details or address the methodological limitations associated with this novel approach. In the present study, we significantly advance the application of the calorimetric FORC method by thoroughly investigating its implementation on a prototypical bulk SCO system. Particular attention is given to the numerical treatment involved in calculating the FORC distributions and to a detailed comparison with results typically obtained via magnetometry. Beyond the experimental analysis, we also propose a novel computational framework for modeling calorimetric FORC-like results within an Ising-like spin system, offering a predictive tool for simulating the complex switching dynamics observed in these materials. This combined experimental and theoretical approach provides a deeper understanding of the calorimetric FORC methodology, its advantages, and its limitations, thereby setting the stage for its broader application in the study of SCO systems.

## 2. Materials and Methods

**Figure 1:**
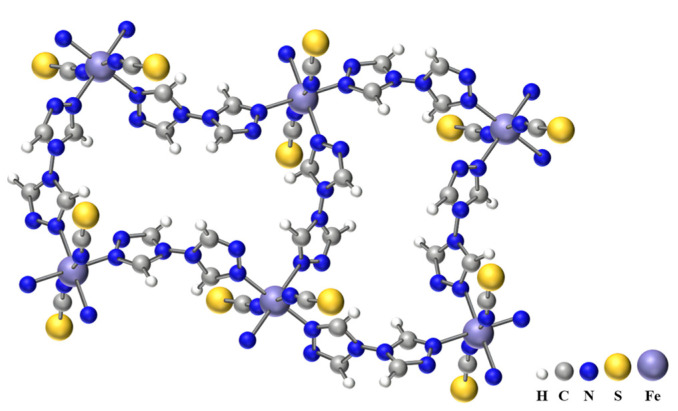
Layered structure of Fe(btr)_2_(NCS)_2_∙H_2_O.

The prototypical SCO compound studied here in bulk form is the 2D coordination polymer [Fe(btr)_2_(NCS)_2_]∙H_2_O (btr = 4,4′-bis(1,2,4-triazole)], which undergoes a full, hysteretic spin transition when cooled below room temperature [29]. The bistability in the hysteretic range is associated with an isostructural phase transition. The crystal structure consists of a stack of planes formed by Fe(II) ions interconnected by bis-triazole bridging ligands. These planes interact with each other via hydrogen bonds (uncoordinated water molecules) and van der Waals interactions (Figure 1). The FeN_6_ coordination sphere includes six nitrogen atoms of two thiocyanate (Fe-N(CS)) bonds (in the apical position) and four equatorial bonds with btr ligands [30]. Synthesis followed the procedure outlined in Ref. [31], and elemental analysis confirmed its analytical purity.

The material’s distinct polymeric layer arrangement fosters significant in-plane cooperative interactions. In the low-spin (LS) state, significant structural modifications compared to the high-spin (HS) state include shorter Fe–N bond lengths [Δd_Fe-NCS_ = −0.175(4) Å, Δd_Fe-N(btr)_ = −0.213(3) Å], a more linear Fe–N–C–S arrangement due to NCS group reorientation, and a 4.8% reduction in the unit cell volume [ΔV = −91(9) Å^3^]. Associated with the HS–LS transition, the material displays thermochromic effects, appearing colorless at room temperature and purple when cooled [32].

A strong ligand field enables the complex’s bistable behavior, with the thermal hysteresis loop serving as evidence of a first-order phase transition. The phase transition results in internal strains that lead to crystal breaking during the LS→HS transition. Fortunately, after undergoing several thermal cycles, the sample reaches a stable state, allowing for reproducible measurements. Typically, a minimum of 20 stabilization loops are conducted prior to initiating the actual measurements [17].

During the transition between two states, all observed changes are reported using a single, universal parameter, n_HS_, which indicates the fraction of molecules in the high-spin state.

## 3. Results and Discussion

### 3.1. Magnetometric Measurements

The vast majority of FORC measurements on spin-crossover compounds have been made using SQUID magnetometry [17,33]. Within this context, FORC experiments involving temperature variations should be categorized based on the direction of the thermal sweep, distinguishing between heating-type and cooling-type protocols, as the sign of the temperature change plays a critical role in the interpretation of the results. To create FORC diagrams, we begin by heating or cooling the system to a temperature where it is entirely in either its HS or LS state, ensuring a uniform initial configuration. Next, the temperature is adjusted (decreased or increased) to a chosen reversal temperature (T_R_ for cooling, T_R_* for heating). From this reversal point, the temperature is then changed in the opposite direction until the system is once again fully saturated in either the HS or LS state. This entire sequence is performed for numerous reversal temperatures. The resulting data, specifically n_HS_(T_R_,T) for cooling and n_HS_(T_R_*,T) for heating, is then transformed to generate the FORC diagrams. Usually, processed data points will be plotted on an evenly spaced grid in the (T,T_R_) coordinate system.

Mathematically, a FORC distribution is determined by calculating the second derivative with respect to two temperatures of system’s measured output,

(1) $$\rho \left(T_{R},T\right)=-\frac{\partial^{2}n_{\mathrm{HS}}\left(T_{R},T\right)}{\partial T_{R}\partial T}\;\mathrm{and}\;\rho \left({T_{R}}^{*},T\right)\;=+\frac{\partial^{2}n_{\mathrm{HS}}\left({T_{R}}^{*},T\right)}{\partial {T_{R}}^{*}\partial T}$$

for heating (i.e., FORC heating) and cooling (i.e., FORC cooling) modes, respectively, while the FORC diagram represent the contour plot of the distribution.

**Figure 2:**
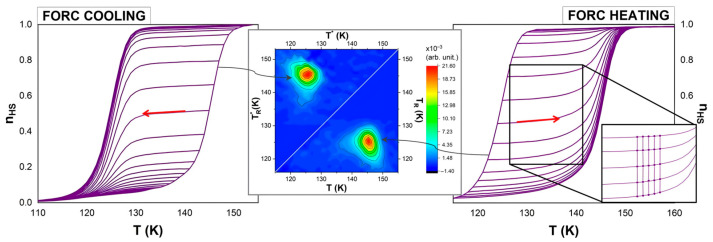
FORC curves and corresponding diagrams from magnetometric measurements of bulk Fe(btr)_2_(NCS)_2_∙H_2_O. The experiments involved temperature sweeping at a rate of 0.3 K min^−1^ during both the cooling and heating processes. The red arrow indicates the direction of change in temperature. Adapted with permission from Ref. [18]. Copyright 2019 American Chemical Society. Inset right panel: a subset of five consecutive heating FORCs where the circled points represent a 5 × 5 array of data in the (T, T_R_) plane.

The FORCs and FORC diagrams measured experimentally (technical details are provided in the Appendix A) for the coordination polymer [Fe(btr)_2_(NCS)_2_]∙H_2_O] are presented in Figure 2, both for heating and cooling modes. To easily compare the FORC distributions, we represent them in the two half planes, separated by the T_R_ = T_R_* = T line, with the heating diagram below and the cooling diagram above this line.

The key challenge in constructing FORC diagrams is the accurate evaluation of the second-order derivative of n_HS_, the fraction of molecules in the high-spin (HS) state, as any noise inherently present—even if not immediately apparent in the original dataset—becomes significantly amplified during this process. To address this, Pike [34] proposed calculating the FORC distribution on a (T, T_R_) grid by employing a local square lattice of data points. The number of points on the local lattice is given by (2 SF + 1)^2^, with SF representing the configurable smoothing factor, which can be set at 2 for well-behaved samples or higher for samples with low signal-to-noise ratios [35]. For SF = 2, smoothing is performed across a 5 × 5 array of data (see Figure 2, inset). Next, the n_HS_ fraction at these data points is fitted with a polynomial surface in a least-squares manner (standard FORC method) and the coefficient of the mixed term is the FORC distribution at point P corresponding to the center of the lattice [34]. One limitation of this approach is the potential loss of fine structural details when applied to noisy datasets, which necessitate the use of a high smoothing factor to suppress noise. Additionally, the resulting diagram will omit the first and last *n* curves (*n* = SF), which could result in a trimmed picture.

**Figure 3:**
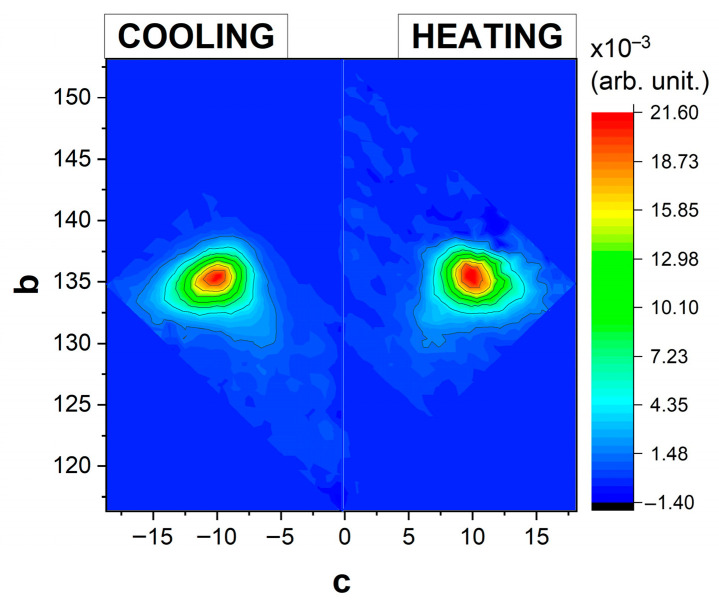
FORC diagrams represented in (coercivity, bias), i.e., (c, b) coordinates for bulk Fe(btr)_2_(NCS)_2_∙H_2_O from magnetometric measurements. The coercivity is related to (T, T_R_) through the relation $c=\left(T-T_{R}\right)/2$, while the bias is given by $b=\left(T+T_{R}\right)/2$. These transformations only modify the coordinate system in which the diagram is represented, while the distribution itself remains unchanged. To plot both cooling and heating modes on the same graph, negative coercivity values were conventionally assigned to the cooling mode diagram.

FORC diagrams plotted in the temperature plane (T, T_R_) do not directly reveal the physical properties essential for characterizing spin transition materials. A more informative approach is to transform these diagrams into a physical parameters plane, using (coercivity, bias) coordinates, as shown in Figure 3. These new coordinates approximate the energy gap Δ and the intra-domain interaction J [17].

### 3.2. Calorimetric Measurements

Calorimetric measurements are used as a complementary method to provide a quantitative analysis of the spin-crossover behavior. The differential scanning calorimetry (DSC) quantifies the heat flow linked to the rate at which a spin transition occurs, unlike magnetometric methods, which measure the current proportion of transformed spins (see also Appendix A). DSC is particularly effective at detecting phase transformations of SCO, but moreover, it detects crystallization, melting, and glass transitions of the embedding matrix in case of composite structures [18].

**Figure 4:**
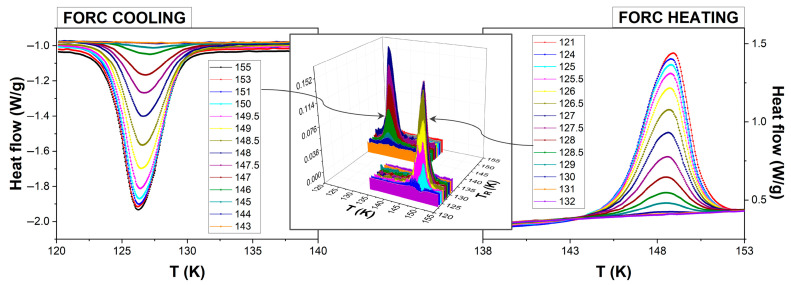
FORC thermograms (sides) and FORC distributions (in the middle) from calorimetric measurements of Fe(btr)_2_(NCS)_2_∙H_2_O bulk polycrystallites (endothermic signal up).

Recording calorimetric FORCs follows the same protocol as recording magnetometric FORCs (described in the previous section), i.e., starting from saturation, sweeping the temperature until a reversal temperature, and then returning to saturation, the only difference being the output parameter, which is not the HS fraction but rather the heat flow (denoted with H(T)). Thus, one obtains the heat capacity profiles, as shown in Figure 4 (side panels), for cooling and heating modes, which are fundamentally different from the typical FORCs (see Figure 2). The classical methodology for calculating the FORC diagram could no longer be applied under these conditions, making it necessary to develop a new approach for processing the experimental data. Fortunately, the calculation of calorimetric FORC diagrams proved to be more straightforward, as the heat flow H(T) already corresponds to the first derivative of n_HS_ with respect to temperature. To obtain the FORC distribution (Figure 4, middle panel), it is sufficient to perform a single derivative of H(T) with respect to the reversal temperature,

(2) $$\rho \left(T,\;T_{R}\right)=-\frac{\partial H\left(T\right)}{{\partial T}_{R}}\;\mathrm{and}\;\rho \left(T,\;{T_{R}}^{*}\right)=+\frac{\partial H\left(T\right)}{\partial {T_{R}}^{*}}$$

for heating and cooling modes, respectively. This derivative can be directly computed by numerically evaluating the change in the measured heat flow with respect to the variations in the reversal temperature, following a finite-difference approximation scheme, without the need for complex interpolation algorithms such as that discussed in the previous section.

**Figure 5:**
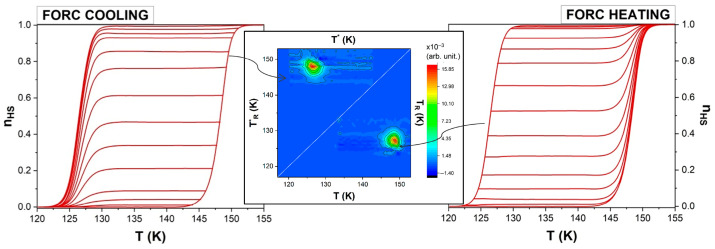
FORC curves and diagrams for Fe(btr)_2_(NCS)_2_∙H_2_O bulk in both heating and cooling modes for calorimetric measurements.

We checked for consistency in data treatment by integrating the thermogram with respect to the temperature. As expected, we obtain the FORC curves and diagrams (Figure 5) similar to the ones from magnetometric measurements (see also Figure 2).

Calorimetric measurements differ subtly but significantly from magnetometric ones in terms of the experimental protocol. A key distinction lies in the relatively large temperature interval between consecutive reversal temperatures—typically ranging from 0.5 to 1 K, as opposed to 0.1–0.2 K in magnetometric experiments. This is primarily due to the requirement that all FORC curves be recorded within a single continuous experimental run, ensuring consistent measurement conditions throughout. Hence, the number of consecutive FORC curves measured in a single experiment (ca. 15–20 when applying a temperature ramp of 1 K/min) is determined by the limited autonomy duration in the liquid nitrogen of the cooling system. It is also important to use a sufficiently small temperature increment at each fixed reversal temperature to ensure well-resolved and smooth curves, which, as previously discussed, correspond to the first derivative of the high-spin fraction. Therefore, the grid for FORC evaluation based on polynomial fitting becomes unevenly spaced, which can introduce artificial features into the diagram. Additionally, the omission of the first and last smoothing factor (SF) curves—as discussed in the previous section—may lead to incomplete or truncated diagrams. These limitations are easily avoided in the calorimetric measurements, where the FORC diagram can be constructed using only a single derivative, thereby reducing sensitivity to grid spacing and smoothing-related artifacts. Moreover, we evaluated the noise level in the calorimetric FORC by analyzing its degree of smoothness and found it to be at least one order of magnitude lower than that of the magnetometric FORC, in the absence of any additional polynomial fitting.

### 3.3. Simulations

Building on the experimental results, we now introduce numerical simulations using an Ising-like model to both validate and provide deeper insight into the observed phenomena. One of the key advancements presented here is the implementation of a computational protocol that directly generates thermograms from the model.

**Figure 6:**
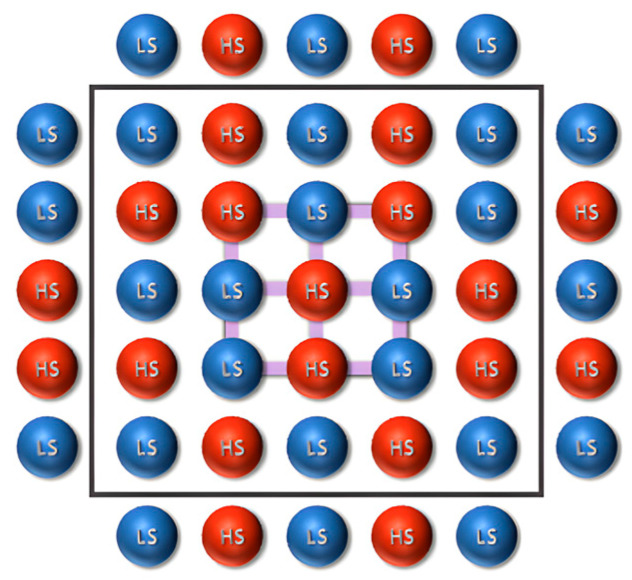
An Ising-like network with periodic boundary conditions, containing HS (red) and LS (blue) molecules.

The building block in the Ising model adapted to the specificities of spin crossover is the fictitious spin (σ_i_ = +1 for HS state and σ_i_ = −1 for LS) linked to each molecule arranged in a rectangular planar network with periodical boundary conditions (see Figure 6) being described by a Hamiltonian accounting for short- and long-range intermolecular interactions and a temperature-dependent field:

(3) $$H=\frac{1}{2}\sum_{i}\left(∆-k_{B}T\;\ln(g)\right)\sigma_{i}-J\sum_{i,j}\sigma_{i}\sigma_{j}-G\sum_{i}\sigma_{i}<\sigma >,$$

with Δ being the energy difference, $k_{B}$ being Boltzmann constant, T being the system temperature, $g=\frac{g_{HS}}{g_{LS}}$ being the degeneracy ratio between the two states related to the entropy ∆S=lng, J being the short-range interaction, and G being the long-range interaction parameters. These parameters, i.e., J and G, are purely phenomenological, representing the elastic properties of the spin-crossover complex. It is important to note that these parameters should not be interpreted in analogy with the exchange interactions characteristic of ferromagnetic systems. Moreover, the values considered for the fictitious spins aim to simplify the mathematical and computational treatment of the system by reflecting the binary nature of the spin transition, though it is not related to the actual spin quantum numbers of the metal centers. Since our primary focus is on simulating the thermograms, we simplify the Hamiltonian presented in Equation (3) by neglecting long-range interactions. In this simplified version of the model, only short-range interactions are considered, disregarding the last term in Equation (3).

A molecule can change its state between LS and HS probabilistically (see Equation (4)) as dictated by Arrhenius dynamics [33], which basically assumes the existence of an energy barrier E_A_. Moreover, the short-range interaction acts only on the first-order neighbors, mathematically reflected by the sum $\sum \sigma_{neighbors}$, and τ is a scaling constant.

(4) $$\left\{\begin{array}{c} P_{\mathrm{HS}\;\to \;\mathrm{LS}}=\frac{1}{\tau }\;e^{\frac{∆-k_{B}T∆S}{2k_{B}T}}\;e^{-\;\frac{E_{A}+2J\;\sum \sigma_{\mathrm{neighbors}}}{k_{B}T}}\\ P_{\mathrm{LS}\;\to \;\mathrm{HS}}=\frac{1}{\tau }\;e^{-\;\frac{∆-k_{B}T∆S}{2k_{B}T}}\;e^{-\;\frac{E_{A}-2J\;\sum \sigma_{\mathrm{neighbors}}}{k_{B}T}} \end{array}\right.$$

The system properties are calculated based on a standard Monte Carlo algorithm [33], which considers that probabilities are computed sequentially only for randomly selected molecules, which will switch if the transition probability is bigger than a fresh random number r ϵ [0, 1]. This procedure is repeated for a considerable number of times until the system’s true equilibrium state is reached. Based on previous studies [33], we assume that the set of parameters associated to each molecule are not the same but rather slightly different. In our simulations the parameters are Gaussian-distributed.

**Figure 7:**
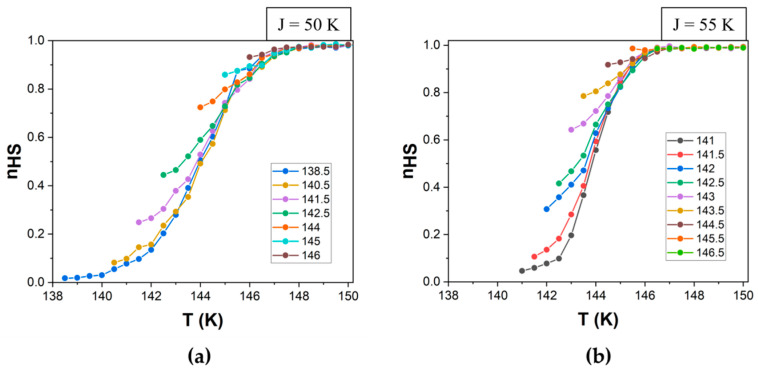
Simulated magnetometric FORCs in the heating mode for ΔS = 7, E_A_ = 400 K, k_B_ = 1, μ_Δ_ = 1000, with σ_Δ_ = 200 and (**a**) J = 50 and (**b**) J = 55. Lines are guides for the eyes. The reversal temperatures are indicated in the legend for each panel.

Using this method, we can calculate the value of the high-spin fraction at any temperature (Figure 7) based on a fictitious spin average value. Since the average spin <σ> ranges from −1 (corresponding to a system composed entirely of LS molecules) to +1 (entirely HS molecules), a linear transformation is used to map this range onto the interval [0, 1]. Specifically, the high-spin fraction is given by $n_{\mathrm{HS}}=\left(1+<\sigma >\right)/2$, where $n_{\mathrm{HS}}=0\;$ corresponds to the pure LS state and $n_{\mathrm{HS}}=1\;$ to the pure HS state. Alongside this standard result of the simulation, given that the heat flow (ΔH) is proportional to the number of molecules that switch their spin state at each temperature (Δn = n_HS→LS_ − n_LS→HS_), we propose for the first time computing the thermogram based on Equation (5).

(5) $$∆H=∆H_{\mathrm{LS}\;\to \;\mathrm{HS}}-∆H_{\mathrm{HS}\;\to \;\mathrm{LS}}\;\sim \;∆n$$

**Figure 8:**
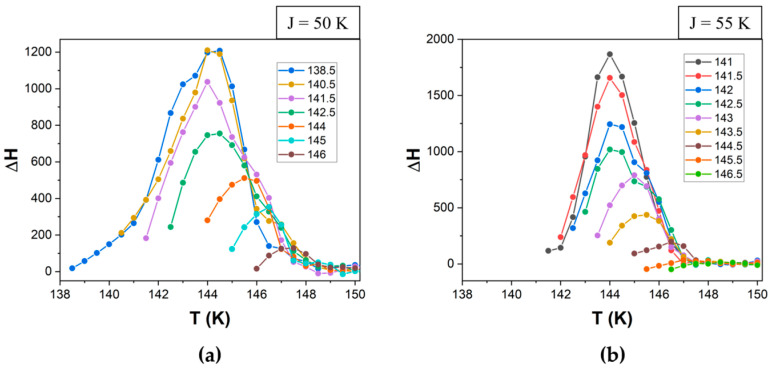
Simulated calorimetric data (thermograms) in the heating mode for ΔS = 7, E_A_ = 400 K, k_B_ = 1, μ_Δ_ = 1000, with σ_Δ_ = 200 and (**a**) J = 50 and (**b**) J = 55. Lines are guides for the eyes. The reversal temperatures are indicated in the legend for each panel.

The simulation parameters for a quadratic system consisting of 100 × 100 particles with periodic boundary conditions were as follows: ΔS = 7, E_A_ = 400 K, k_B_ = 1 and the Gaussian distributions for Δ, centered (μ_Δ_) to 1000, with a standard deviation (σ_Δ_) of 200 and constant values for J = 50 K (Figure 7a and Figure 8a) or 55 K (Figure 7b and Figure 8b).

**Figure 9:**
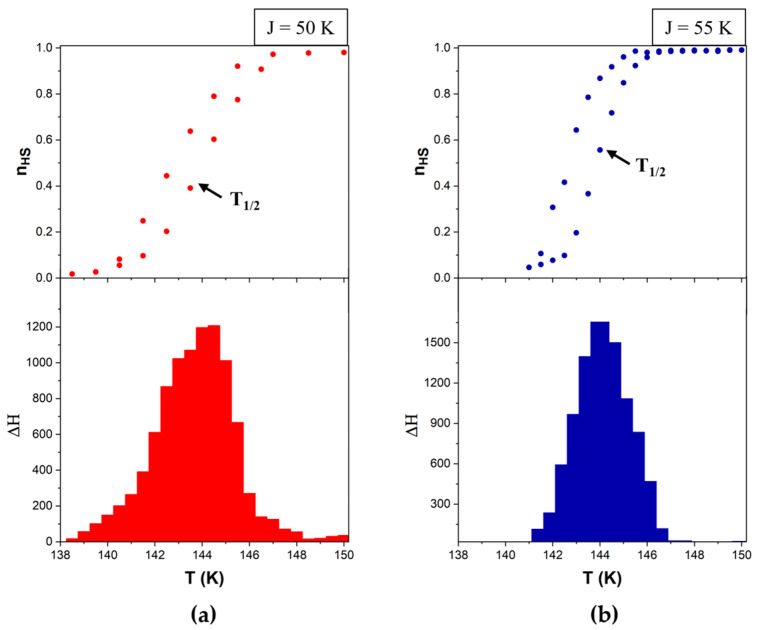
Comparison between a major hysteresis loop (magnetometric data—up) and a heat capacity profile, simulated in heating mode (calorimetric data—down) for ΔS = 7, E_A_ = 400 K, k_B_ = 1, μ_Δ_ = 1000, with σ_Δ_ = 200 and (**a**) J = 50 and (**b**) J = 55.

To further analyze the calorimetric method in comparison with the classical magnetometric approach, we represented a magnetic hysteresis loop and its corresponding calorimetric simulation. As we can see in Figure 9, the calorimetric peak is at the point where the high-spin fraction undergoes a sharp rise, i.e., at T_1/2_ (equilibrium temperature—the temperature at which the populations of high-spin and low-spin molecules are the same—n_HS_ = 0.5).

**Figure 10:**
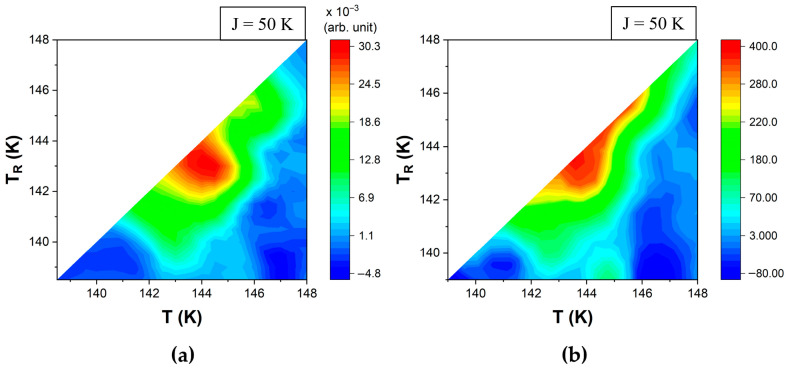
Simulated calorimetric-type FORC diagrams in the heating mode for ΔS = 7, E_A_ = 400 K, k_B_ = 1, μ_Δ_ = 1000, with σ_Δ_ = 200 and J = 50 (**a**) using the standard FORC method, and (**b**) by calculation from thermogram data. The color bar scales in panels (**a**,**b**) differ by several orders of magnitude as the double mixed derivative (panel (**a**)) is based on the n_HS_ fraction, which spans from 0 to 1, while the single derivative (panel (**b**)) is based on ΔH, which is proportional to the variation in the number of spins.

**Figure 11:**
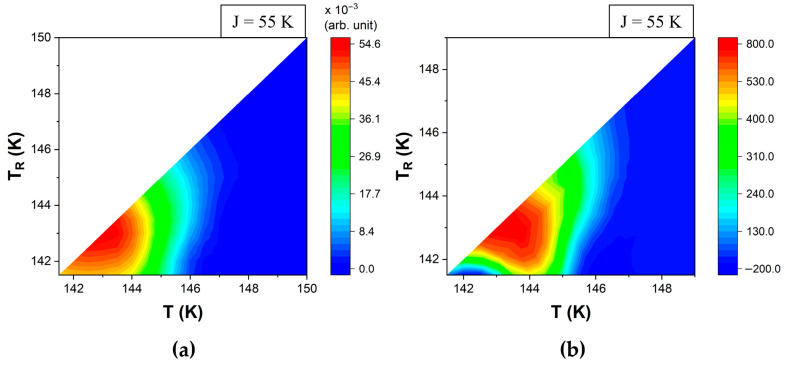
Simulated calorimetric-type FORC diagrams in the heating mode for ΔS = 7, E_A_ = 400 K, k_B_ = 1, μ_Δ_ = 1000, with σ_Δ_ = 200 and J = 55 (**a**) using the standard FORC method and (**b**) by calculation from thermogram data. The color bar scales in panels (**a**,**b**) differ by several orders of magnitude as the double mixed derivative (panel (**a**)) is based on the n_HS_ fraction, which spans from 0 to 1, while the single derivative (panel (**b**)) is based on ΔH, which is proportional to the variation in the number of spins.

The (n_HS_, T) data was plotted as FORC diagrams using the polynomial fitting (i.e., standard FORC method) for smoothing factor *SF* = 3, i.e., 49 nearest neighbors for each input point (Figure 10a and Figure 11a).

FORC diagrams were also obtained from calorimetric data by calculating the derivative of the smoothed values of the heat flow (Figure 10b and Figure 11b). The similarities between these FORC diagrams provide a strong argument in favor of the approach proposed here to evaluate FORC thermograms. Although simulations allow the flexibility to choose between both methods, for the experimental data, only the direct calculation of the derivative should be used.

## 4. Conclusions

In this paper, we have explored two complementary measuring techniques for characterizing the spin-crossover compound [Fe(btr)_2_(NCS)_2_]·H_2_O using the FORC method. For magnetometry, the FORC calculation requires a surface polynomial fitting, whereas in calorimetry, just one derivative fully determines the FORC distribution. Both methods lead to the same results. More broadly, the calorimetric approach simplifies diagram calculation when the reversal temperature step between successive cycles significantly differs from the measurement step within a single cycle. The Ising-like model successfully reproduces both experiments, and an original method to calculate the thermograms is introduced. This study has broader implications for composite materials research, where calorimetry not only effectively differentiates between various phases but also plays a key role in shaping the development and design of next-generation bistable SCO materials for cutting-edge applications in nanotechnology.

In addition to the clear efficiency and added value this method offers in the field of spin-crossover materials, it also serves as a valuable experimental tool in the calorimetric studies of giant magnetocaloric effects in various materials [36,37]. Furthermore, the single-derivative approach used to calculate the FORC distribution is particularly well-suited for soft magnetic materials, where the output signal in inductometric measurements is already the time-dependent derivative of the magnetic induction with respect to the applied field. This inherent compatibility simplifies the implementation of FORC analysis in such systems, eliminating the need for further signal processing.

## Data Availability

The original contributions presented in this study are included in the article. Further inquiries can be directed to the corresponding author.

## Abbreviations

The following abbreviations are used in this manuscript:

FORC	First-Order Reversal Curve
SQUID	Superconducting Quantum Interference Device
DSC	Differential Scanning Calorimetry
SCO	Spin Crossover
HS	High Spin
LS	Low Spin

## Appendix A

### Appendix A.1. Calorimetric Measurements

A Q-20 device (TA Instruments, New Castle, DE, USA), incorporating a liquid nitrogen cooling module, was used for differential scanning calorimetry measurements. Calibration of temperature and heat flux relied on the melting transition of an indium sample. A weighted mass m = 12.38 mg of spin-crossover complexes was loaded in an aluminum hermetic pan. FORC cycles were recorded at a constant rate of 1 K/min. In order to acquire a complete set of FORC cycles in both heating and cooling modes, the reversal temperatures (T_R_/T_R_*) corresponding to two subsequent cycles were respectively increased or decreased by a step of typically 0.5 to 1 K.

### Appendix A.2. Magnetometric Measurements

A MPMS XL (MPMS—Magnetic Properties Measurement System) SQUID device (Quantum Design, San Diego, CA, USA) was used to record the magnetic signal of the spin-crossover sample under a constant applied magnetic field of 5000 Oe, following standard calibration against a palladium reference. FORC curves were measured using the ‘‘settle’’ mode of the temperature control module, with an average temperature sweep rate of 0.3 K/min, to ensure thermal stabilization and minimize kinetic effects. The $n_{HS}$ fraction was derived from the magnetic susceptibility χ using following relation:

$$n_{HS}=\frac{\chi T-{\left(\chi T\right)}_{LS}}{{\left(\chi T\right)}_{HS}-{\left(\chi T\right)}_{LS}}$$

where ${\left(\chi T\right)}_{LS}$ and ${\left(\chi T\right)}_{HS}$ correspond to saturated LS and HS states, respectively. This expression encapsulates several properties of the SCO materials, such as that the LS state is diamagnetic (magnetic signal does not change with temperature), the HS state is paramagnetic (χ is described by the Curie–Weiss law), and the applied field is constant and low enough to remain within the linear magnetic regime.

## References

[1] Gütlich P., Goodwin A. (2004). Spin Crossover in Transition Metal Compounds.

[2] Halcrow M.A. (2013). Spin-Crossover Materials—Properties and Applications.

[3] Xu F.-X., Zhang X.-Y., Wei H.-Y., Wang X.-Y. (2024). Hysteretic spin crossover in a Hofmann-type metal–organic framework constructed from a [MoIII(CN)7]^4−^ unit. Inorg. Chem. Front..

[4] Zhao X.-H., Deng Y.-F., Xi J., Huang J.-Q., Zhang Y.-Z. (2025). Supramolecular Spring-Like Fe(II) Spin-Crossover Complexes Experiencing Giant and Anisotropic Thermal Expansion Across Two Distinct Temperature Regimes. Angew. Chem.-Int. Edit..

[5] Linares J., Codjovi E., Garcia Y. (2012). Pressure and Temperature Spin Crossover Sensors with Optical Detection. Sensors.

[6] Raza Y., Volatron F., Moldovan S., Ersen O., Huc V., Martini C., Brisset F., Gloter A., Stephan O., Bousseksou A. (2011). Matrix-dependent cooperativity in spin crossover Fe(pyrazine)Pt(CN)4 nanoparticles. Chem. Commun..

[7] Tokarev A., Long J., Guari Y., Larionova J., Quignard F., Agulhon P., Robitzer M., Molnár G., Salmon L., Bousseksou A. (2013). Spin crossover polysaccharide nanocomposites. New J. Chem..

[8] Salmon L., Catala L. (2018). Spin-crossover nanoparticles and nanocomposite materials. Comptes Rendus Chim..

[9] Manrique-Juárez M.D., Suleimanov I., Hernández E.M., Salmon L., Molnár G., Bousseksou A. (2016). In Situ AFM Imaging of Microstructural Changes Associated with The Spin Transition in [Fe(Htrz)2(Trz)](Bf4) Nanoparticles. Materials.

[10] Sy M., Traiche R., Fourati H., Singh Y., Varret F., Boukheddaden K. (2018). Spatiotemporal Investigations on Light-Driven High-Spin-Low-Spin Interface Dynamics in the Thermal Hysteresis Region of a Spin-Crossover Single Crystal. J. Phys. Chem. C.

[11] Tanasa R., Stancu A., Codjovi E., Linares J., Varret F., Létard J.-F. (2008). A first order reversal curve investigation of pressure hysteresis in multiferroics spin transition compound. J. Appl. Phys..

[12] Béron F., Novak M.A., Vaz M.G.F., Guedes G.P., Knobel M., Caldeira A., Pirota K.R. (2013). Macroscopic quantum tunneling of magnetization explored by quantum-first-order reversal curves. Appl. Phys. Lett..

[13] Franco V., Gottschall T., Skokov K.P., Gutfleisch O. (2016). First-Order Reversal Curve (FORC) Analysis of Magnetocaloric Heusler-Type Alloys. IEEE Magn. Lett..

[14] Belyaev V.K., Murzin D., Martínez-García J.C., Rivas M., Andreev N.V., Kozlov A.G., Samardak A.Y., Ognev A.V., Samardak A.S., Rodionova V. (2021). FORC-Diagram Analysis for a Step-like Magnetization Reversal in Nanopatterned Stripe Array. Materials.

[15] Arzuza L.C., Beron F., Pirota K.R. (2021). High-frequency GMI hysteresis effect analysis by first-order reversal curve (FORC) method. J. Magn. Magn. Mater..

[16] Cabanas A.M., Pérez del Real R., Laroze D., Vázquez M. (2023). First-Order Reversal Curves of Sets of Bistable Magnetostrictive Microwires. Materials.

[17] Tanasa R., Enachescu C., Stancu A., Linares J., Codjovi E., Varret F., Haasnoot J.G. (2005). First-order reversal curve analysis of spin-transition thermal hysteresis in terms of physical-parameter distributions and their correlations. Phys. Rev. B.

[18] Tanasa R., Enachescu C., Laisney J., Morineau D., Stancu A., Boillot M.L. (2019). Unraveling the Environment Influence in Bistable Spin-Crossover Particles Using Magnetometric and Calorimetric First-Order Reverse Curves. J. Phys. Chem. C.

[19] Tanasa R., Enachescu C., Stancu A., Varret F., Linares J., Codjovi E. (2007). Study of impurities effect in spin crossover compounds using first order reversal curves (FORC) method. Polyhedron.

[20] Boukheddaden K., Ritti M.H., Bouchez G., Sy M., Dirtu M.M., Parlier M., Linares J., Garcia Y. (2018). Quantitative Contact Pressure Sensor Based on Spin Crossover Mechanism for Civil Security Applications. J. Phys. Chem. C.

[21] Tanasa R., Stancu A., Létard J.F., Codjovi E., Linares J., Varret F. (2007). Piezo- and thermo-switch investigation of the spin-crossover compound [Fe(PM-BiA)2(NCS)2]. Chem. Phys. Lett..

[22] Rat S., Piedrahita-Bello M., Salmon L., Molnár G., Demont P., Bousseksou A. (2018). Coupling Mechanical and Electrical Properties in Spin Crossover Polymer Composites. Adv. Mater..

[23] Piedrahita-Bello M., Angulo-Cervera J.E., Enriquez-Cabrera A., Molnár G., Tondu B., Salmon L., Bousseksou A. (2021). Colossal expansion and fast motion in spin-crossover@polymer actuators. Mater. Horiz..

[24] Glazyrin K., Khandarkhaeva S., Dubrovinsky L., Sprung M. (2020). Revisiting spin-state crossover in (MgFe)O by means of high-resolution x-ray diffraction from a single crystal. Phys. Rev. B.

[25] Sorai M., Burriel R., Westrum E.F., Hendrickson D.N. (2008). Mechanochemical effect in the iron(III) spin crossover complex [Fe(3-MeO-salenEt2]PF6 as studied by heat capacity calorimetry. J. Phys. Chem. B.

[26] Benmansour S., Triki S., Gómez-García C.J. (2016). A Spin Crossover Transition in a Mn(II) Chain Compound. Magnetochemistry.

[27] Laisney J., Morineau D., Enachescu C., Tanasa R., Riviere E., Guillot R., Boillot M.L. (2020). Mechanical-tuning of the cooperativity of SC particles via the matrix crystallization and related size effects. J. Mater. Chem. C.

[28] Sorai M., Nakano M., Miyazaki Y. (2006). Calorimetric investigation of phase transitions occurring in molecule-based magnets. Chem. Rev..

[29] Vreugdenhil W., Van Diemen J.H., De Graaff R.A.G., Haasnoot J.G., Reedijk J., Van Der Kraan A.M., Kahn O., Zarembowitch J. (1990). High-spin α low-spin transition in [Fe(NCS)2(4,4′-bis-1,2,4-triazole)2](H2O). X-ray crystal structure and magnetic, mössbauer and EPR properties. Polyhedron.

[30] Martin J.P., Zarembowitch J., Dworkin A., Haasnoot J.G., Codjovi E. (1994). Solid-State Effects in Spin Transitions: Influence of Iron(II) Dilution on the Magnetic and Calorimetric Properties of the Series [FexNi1-x(4,4′-bis(1,2,4-triazole))2(NCS)2].cntdot.H2O. Inorg. Chem..

[31] Vreugdenhil W., Gorter S., Haasnoot J.G., Reedijk J. (1985). Spectroscopic and magnetic properties of a new class of two-dimensional bitriazole compounds: The X-ray structure of poly-bis (thiocyanato-N)-bis-μ-(4,4′-bis-1,2,4-triazole-N1,N1′)-cobalt (II) monohydrate. Polyhedron.

[32] Legrand V., Pillet S., Carbonera C., Souhassou M., Létard J.-F., Guionneau P., Lecomte C. (2007). Optical, Magnetic and Structural Properties of the Spin-Crossover Complex [Fe(btr)2(NCS)2]·H2O in the Light-Induced and Thermally Quenched Metastable States. Eur. J. Inorg. Chem..

[33] Plesca D., Railean A., Tanasa R., Stancu A., Laisney J., Boillot M.L., Enachescu C. (2021). Unexpected Light-Induced Thermal Hysteresis in Matrix Embedded Low Cooperative Spin Crossover Microparticles. Magnetochemistry.

[34] Pike C.R., Roberts A.P., Verosub K.L. (1999). Characterizing interactions in fine magnetic particle systems using first order reversal curves. J. Appl. Phys..

[35] Pike C.R., Roberts A.P., Verosub K.L. (2001). First-order reversal curve diagrams and thermal relaxation effects in magnetic particles. Geophys. J. Int..

[36] Palacios E., Burriel R., Zhang C.L. (2021). Calorimetric study of the giant magnetocaloric effect in (MnNiSi)_(0.56)(FeNiGe)_(0.44). Phys. Rev. B.

[37] Law J.Y., Moreno-Ramírez L.M., Díaz-García Á., Franco V. (2023). Current perspective in magnetocaloric materials research. J. Appl. Phys..

